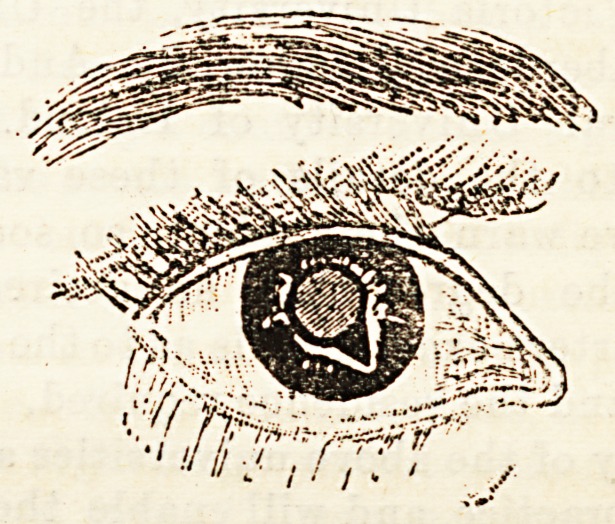# On Modern Progress in Ophthalmic Medicine and Surgery

**Published:** 1895-09-07

**Authors:** Robert Brudenell Carter

**Affiliations:** Consulting Ophthalmic Surgeon to St. George's Hospital


### On Modern Progress in Ophthalmic Medicine and Surgery.

By Robert Brudenell Carter, F.R.C.S., Consulting Ophthalmic Surgeon to St. George's Hospital.

### DISEASES OF THE CORNEA.

Conical Cornea (*continued from page* 319).

An eye which has been treated in the manner described in the last paper should present a well defined patch of corneal opacity, of flattened surface, buff colour, and circular outline, its centre corresponding with what was formerly the apex of the cone. When sufficient time has been given for cicatrisation to be complete, when the marginal parts of the cornea are clear, and when all vascularity arising from the first operation has disappeared, the next step in the management of the case may be undertaken, with a view to the restoration of useful sight. For this purpose the requirement is a lateral or so-called "artificial" pupil.

It is manifest that, when the cornea has been unduly projected forwards and altered in shape, and when its shape has to some extent been restored by the contraction of a central cicatrix, this restoration may be more complete in some portions than in others, and that, other things being equal, the artificial pupil should be placed behind the best portion of cornea that is available for the purpose. For looking at distant objects, the pupil would be equally useful behind any portion of cornea that is not too peripheral and not covered by eyelid; but, for near objects, and for combination of the eyes in a position of convergence, the pupil should be on the nasal side of the eye, and, when possible, in the lower and inner quadrant of the circle. In order to determine the exact position the pupil should be dilated, and the surgeon should carefully study the background of the eye by the direct method of ophthalmoscopy. Where the cornea is least distorted, the view of the retinal vessels will be best defined, and this portion, so long as it is below, or only barely above, the horizontal meridian of the eye, is that which should be selected. The required excision of a piece of iris should be accomplished with great care, and the piece excised should be very small. The best method of proceeding is by what I have formerly described as "optical iridectomy," a full account of which, in relation to laminar cataract, will be found in The Hospital for August 4th, 1894, in the paper from which the appended figure, showing the effect of the operation, is also taken. It will be observed that the opening is V-shaped, and that it does not extend to the corneal margin.

It is essential that the operation for an artificial pupil should not be undertaken as long as there is the slightest remaining irritability of any part of the eye. The iris is very susceptible of the effects of external irritation; and any premature operation on it would probably be followed by inflammation and adhesions. The most disastrous result that I have ever witnessed in a case of conical cornea occurred in the practice of a surgeon who departed from the customary order of procedure. He first made an artificial pupil; and then, before the eye had completely recovered, he scraped and cauterised the cornea. The consequence was an outbreak of iritis of a very severe character, which extended to other tissues, and which eventually led to complete and irrecoverable loss of vision.

Even the tiny wound required for the performance of optical iridectomy is liable, in healing, to bring about some modification of ocular curvature, and on this account it is well to wait for several weeks after the second operation before deciding upon the question of glasses for the improvement of vision. The most successful case will usually display some residual error of refraction, for which lenses will be helpful; and, whenever required, these ought to be given before the eyes are suffered to undertake any work of a trying character. It is essential that the functions of the affected organs should be performed under the most favourable attainable conditions.

The next step, the tattooing of the scar, has reference mainly to appearance, but, unless the scar be absolutely opaque, it will also to some extent improve vision. If light can enter through the cicatrical tissue, it will be diffused light only, from which no retinal images can be formed, but which will have a tendency to diminish the vividness of the images formed by light passing through the pupil. This effect is at once removed by the layer of Indian ink left by tattooing in the superficial layer of the new tissue.

The best tattooing instrument, in my judgment, is a very fine and very sharp bayonet-shaped needle, with a deep groove on one side, and set in a convenient handle. The eye should be cocainised, the lids separated by a speculum, and a morsel of sponge tucked under the margin of the upper lid on the outer side, to absorb tears, and to keep the surface dry. Fixation forceps should be avoided, lest the pigment should find its way into their punctures, and leave indelible marks where none are desired. The eye may be effectually steadied by the fingers, and the outline of the scar should first be traced out by obliquely directed needle punctures, after which the whole of the area should be punctured in a similar manner, the punctures being set as closely together as possible. Sufficient Indian ink, in impalpable powder, is then thoroughly rubbed into the punctures with a fine smooth wooden spatula. The powder is then wiped off with a morsel of moist sponge, and any portion of the scar which has not "taken" the colour is punctured anew and more powder is rubbed in. No dressing or treatment is required after the operation. Any redness or tenderness will subside in a day or two, and after this time nothing but close inspection will reveal the very existence of the scar, while the effect seems to be absolutely permanent, and calculated to last as long as the cornea itself.

## Figures and Tables

**Figure f1:**